# The impact of GJA8 SNPs on susceptibility to age-related cataract

**DOI:** 10.1007/s00439-018-1945-5

**Published:** 2018-10-22

**Authors:** Xiaoning Yu, Xiyuan Ping, Xin Zhang, Yilei Cui, Hao Yang, Xiajing Tang, Yelei Tang, Xingchao Shentu

**Affiliations:** 1 Eye Center, The Second Affiliated Hospital, Zhejiang University School of Medicine, Hangzhou, 310009 Zhejiang China; 2 The Second Affiliated Hospital, Zhejiang University School of Medicine, Hangzhou, 310009 Zhejiang China

## Abstract

The *gap junction protein alpha 8* (*GJA8*) gene has been widely studied in human congenital cataracts. However, little is known about its relationship with age-related cataract (ARC). In this study, three *GJA8*-tagged single nucleotide polymorphisms related to an increased ARC risk were identified: rs2132397 for general ARC under both dominant and additive models; rs7541950 for general ARC under both recessive and additive models; and rs6657114 for cortical cataract under the recessive model. To uncover the underlying mechanisms, this study also sought to explore whether GJA8 is involved in the autophagy process in human lens epithelial cells. The results showed that GJA8 may participate in autophagy to maintain the intracellular environment, which may be a novel mechanism for cataract formation induced by *GJA8*. In conclusion, this study identified the genetic susceptibility of *GJA8* polymorphisms on ARC and provides new clues for fully understanding the pathological mechanism of *GJA8* variants in affecting lens opacity.

**Electronic supplementary material:**

The online version of this article (10.1007/s00439-018-1945-5) contains supplementary material, which is available to authorized users.

## Introduction

Cataracts are the leading cause of blindness and a major cause of vision impairment worldwide (Bourne et al. 2013; Congdon et al. 2003), with age-related cataract (ARC) being the most common type (Klein et al. 1992, Su et al. 2013). The precise etiology of ARC is not yet fully understood, but it is widely accepted that genetic factors play a vital role in the formation of ARC (Su et al. 2013). Studies of twins have implicated that the broad sense heritability is 58% for the cortical subtype of ACR and 48% for the nuclear subtype of ARC (Hammond et al. 2000, 2001). Genetic variations may be directly involved in the development of ARC or may increase lens susceptibility to environmental risk factors (Hammond et al. 2001). To date, over 40 different genes and loci have been identified as related to congenital cataract formation (Mackay et al. 2014; Shiels and Hejtmancik 2013). The connexin genes, which account for a quarter of congenital cataract-related genes, are reported to be among the most widespread (Mackay et al. 2014).

The *Gap junction protein alpha 8* (*GJA8*) gene, which belongs to the connexin gene family, is located at lq21.1 (CX50, MIM 600897, NG_016242.1) and consists of two exons (Dang et al. 2016; Li et al. 2013; Zhu et al. 2014). This gene encodes connexin 50, which is extensively and abundantly distributed in lens fibers and epithelial cells, as well as constituting a highly developed gap junction network vital for maintaining lens transparency (Beyer et al. 2013; Goodenough 1992; Jiang 2010). To date, more than 20 *GJA8* variations have been identified in congenital cataract pedigrees worldwide, and most are inherited through autosomal dominance (Dang et al. 2016; Zhu et al. 2014). However, few studies have reported its relationship with ARC. Additionally, only one *GJA8* variant, c. 823G > A, which was first identified in a congenital cataract pedigree, has been reported to specifically cause this disease (Liu et al. 2011; Patel et al. 2017; Zhou et al. 2011). Thus, although *GJA8* has been extensively investigated in congenital cataracts, further studies should be conducted to explore its relationship with ARC, and the results may be diverse among different ethnicities. Although functional disturbances of gap junctions and hemichannels caused by *GJA8* variants are considered to be the main pathogenesis for cataract formation (Beyer et al. 2013; Goodenough 1992), Lichtenstein et al. reported the accumulation of mutated GJA8 proteins resulting from insufficient autophagy in HeLa cells (Lichtenstein et al. 2011), and this study speculates that this may be another pathogenic mechanism of *GJA8* mutations.

Considering that tag single nucleotide polymorphisms (SNPs) make it possible to identify genetic variation and association to phenotypes without genotyping every SNP in a gene, this study aimed to fully screen *GJA8*-tagged SNPs in ARC patients. As the alteration of the autophagy-related proteins LC3 and P62 in GJA8-overexpressed human lens epithelial (HLE) cells was detected, this study also explores whether GJA8 participates in the autophagy process in the lens.

## Materials and methods

### Study participants

A total of 1190 unrelated participants, comprising 690 ARC patients and 550 control subjects, were included in this study. All participants were Han Chinese and were recruited from the Eye Center of the Second Affiliated Hospital, Medical College of Zhejiang University, Hangzhou, China. This study adhered to the tenets of the Declaration of Helsinki and was approved by the ethics committee of the Second Affiliated Hospital, Medical College of Zhejiang University, Hangzhou, China. In addition, informed consent was obtained from every participant.

All ARC patients underwent complete ophthalmic examinations, including fundus photography, the best-corrected visual acuity (BCVA) measurement, and lens examination with a slit lamp biomicroscope after mydriasis. Clinical diagnosis and classification were based on the lens opacities classification system II (Locs II) (Chylack et al. 1989). According to the degenerative regions of the lens, ARC was classified into four subtypes: cortical cataract (*C*), nuclear cataract (*N*), posterior subcapsular cataract (PSC), and multiple cataract (*M*, more than one cataract subtype in an eye) (Klein et al. 1992). Patients with cataracts caused by trauma, uveitis, diabetes, high myopia, and other causes were excluded.

Control subjects were recruited from healthy individuals who received routine health examinations at the Second Affiliated Hospital, Medical College of Zhejiang University, Hangzhou, China. All control subjects also underwent complete ophthalmic evaluations, which indicated transparent lens.

### SNP selection and genotyping

Haplotype-tagging SNPs in the *GJA8* gene were selected from the HapMap Beijing Han Chinese (CHB) population (HapMap Genome Browser release #27, accessed April 29, 2014; available at http://hapmap.ncbi.nlm.nih.gov/). Based on the tagger-pairwise method, with an *R* square (*r*^2^) > 0.8 and a minor allele frequency (MAF) > 0.10, a total of 8 SNPs in *GJA8* were selected. Genomic DNA was extracted from peripheral blood leukocytes from all subjects using a Simgen Blood DNA mini kit (Simgen, Hangzhou, China).

### Statistical analysis

The Hardy–Weinberg equilibrium (HWE) of each SNP was assessed by the *χ*^2^ test using PLINK (v1.07) (available at http://pngu.mgh.harvard.edu/~purcell/plink/). All continuous variables corresponding to the subjects’ characteristics were summarized as mean ± SD. The association between the SNPs and ARC was examined under three different genetic models: dominant, recessive, and additive. Allelic distributions were compared between the ARC patients and the control subjects using the *χ*^2^ test. A logistic regression analysis was conducted to evaluate the genetic effects of the *GJA8* SNPs after adjusting for age and sex. The Bonferroni correction for multiple testing was used to reduce the rate of type I error (We have tested 57 different SNPs in the same case and control groups in all. And GJA8 tag SNPs are only part of them, thus the corrected significant level was set to be 0.05/57*3). An Armitage trend test was performed for the risk SNPs identified by the logistic regression analysis in the additive model using SAS software. All the other statistical analyses were conducted using SPSS software, version 11.0. A two-tailed *p* value < 0.05 was considered statistically significant, otherwise indicated.

### Construction of plasmids

The human *GJA8* coding sequence was acquired by the polymerase chain reaction (PCR) with the primers: sense primer 5′-CTCGAGATATGGGCGACTGG-3′, and antisense primer 5′- CAGAATTCTCATACGGTTAG-3′. The PCR products and vector pEGFP-C1 were digested by *Xho*I and *Eco*rI (Takara, Japan). The construction was confirmed through direct sequencing. Transient transfection of pEGFP-C1-GJA8 was carried out using the Lipofectamine 2000 reagent (Invitrogen, Carlsbad, CA, USA) according to the manufacturer’s protocols.

### Cell culture

The HLE cells (SRA 01-04) (Ibaraki et al. 1998) were obtained from the RIKEN Cell Bank (Tsukuba, Japan) and cultured in DMEM/F12 with 10% heat-inactivated fetal calf serum (Biological Industries, Israel) at 37 °C in a humidified atmosphere containing 5% CO_2_. All cell culture medium components were purchased from Invitrogen Life Technologies. Cells were prepared, and washed with PBS twice, then replaced with no fetal calf serum medium for 2 h or 4 h as was done for the Sta2h group or Sta4h group, or replaced with complete medium for 2 h as was done for the NC group.

### Immunofluorescence

HLE cells were adhered overnight in 24-well plates to grow to 80% cell density, then transfection with or without 1.0 μg/well peGFP-C1-GJA8 plasmid for 24 h, and washed with PBS for twice, replaced with no fetal calf serum media for 2 h or 4 h (Sta2h/Sta4h group) or complete medium (NC group) for 2 h. Then the cells were washed with PBS for twice, and fixed with 4% paraformaldehyde in PBS for 10 min, washed with PBS for three times. Then, cells were blocked with 2% BSA in PBST (0.1% Triton X-100 in PBS) for 1 h and incubated with primary antibody for 2 h [GJA8(sc-50432) 1:200 from Santa Cruz, LC3(2775) 1:200 from Cell Signaling Technology], and washed with PBST for triple; second antibody for 1 h (Alexa Fluor488-conjugated antibodies, Alexa Fluor555-conjugated antibodies, and DAPI from Life Technologies), and washed with PBST for triple. Images were captured using an Olympus FluoView 1000 confocal microscope at 40 ×.

The GFP plasmid vector was used to test the possible alterations of the autophagic proteins in the IF, and no differences between in the GFP plasmid vector and the normal control were observed (Supplementary Figure S1). Thus, in our paper, we use the normal group (the NC group) as the control to explain the alteration of autophagic proteins with the overexpression of GJA8, and to prove that the autophagy process occurred in human lens epithelial (HLE) cells.

### Western blot

HLE cells were adhered overnight in 6-well plates to grow to 80% cell density, then transfection with or without 1.0 or 2.0 μg/well peGFP-C1-GJA8 plasmid for 24 h, and washed with PBS for twice, replaced with no fetal calf serum media for 2 h or 4 h (Sta2h/Sta4h group) or complete medium (NC group) for 2 h. Then the cells were lysed in lysis buffer (Shenggong, Shanghai, China) and blocked in 5% BSA (Shenggong, Shanghai, China) in TBST (0.1% Tween-20 in TBS). Immunocomplexes were separated by 10% SDS-PAGE (sodium dodecyl sulfate-polyacrylamide gel electrophoresis) and then subjected to immunoprecipitation with the primary antibody [P62 (ab56416) 1:1000, α-Tubulin (ab4074) 1:1000 from Abcam], and the HPR-conjugated second antibody.

## Results

### Demographic characteristics of the participants

A total of 690 ARC patients (*C* = 131, *N* = 126, PSC = 73, *M* = 360 cataract patients) and 500 healthy control subjects were included in this study. No significant differences were detected between the two groups (*p* > 0.05). The general demographic characteristics of the 1190 participants are summarized in Table 1.

**Table 1:** The general demographic characteristics of the subjects involved in this study

Group	Number	Gender		Age	
		Male (%)	Female (%)	Mean ± SD	Range
Control	500	58.8	41.2	64.306 ± 7.586	49–92
*ARC*	690	47.83	52.17	66.323 ± 9.781	43–91
*C*	131	34.35	65.65	67.75 ± 8.383	43–88
*N*	126	38.10	61.90	68.259 ± 9.856	45–87
*PSC*	73	45.21	54.79	66.484 ± 10.274	45–90
*M*	360	56.11	43.89	65.170 ± 9.936	38–91

*ARC* age-related cataract, *C* cortical cataract, *N* nuclear cataract, *PSC* posterior subcapsular cataract, *M* multiple cataract

### The bioinformatics characteristics of tag SNPs

Eight tag SNPs in *GJA8* were selected for genotyping; their bioinformatics characteristics are summarized in Table 2. No SNPs deviated from the HWE in this study.

**Table 2:** The bioinformatics characteristics of the involved eight *GJA8* SNPs

SNPs	Minor allele	Call rate	MAF	Test for HWE (*p* value)	Control (MAF)	ARC (MAF)
rs6657114	T	0.99916	0.360	0.6317	0.326	0.385
rs6688578	C	0.99916	0.108	0.4799	0.096	0.118
rs7544630	C	0.99916	0.235	0.4026	0.243	0.229
rs1532399	A	0.99916	0.319	0.6951	0.288	0.342
rs2132397	G	0.99916	0.176	0.3105	0.14	0.202
rs7541950	T	0.99916	0.474	0.1405	0.43	0.493
rs9437981	G	0.99916	0.359	0.7941	0.327	0.382
rs6674829	G	0.99916	0.415	0.1275	0.385	0.436

### The association between tag SNPs and risk of ARC

Two tag SNPs (rs2132397, *p*_a_ = 9.25 × 10^−3^, OR 1.413, CI 1.195–1.671, *p* for the Armitage trend test: 3 × 10^−4^; rs7541950, *p*_a_ = 0.03, OR 1.543, CI 1.230–1.935, *p* for the Armitage trend test: < 1 × 10^−4^) showed a significant association with ARC risk under the additive model (Table 3). One tag SNPs (rs2132397, *p*_a_ = 0.029, OR 1.638, CI 1.267–2.118) showed a significant association with ARC risk under the dominant model, and one tag SNPs (rs7541950, *p*_a_ = 2.33 × 10^−4^, OR 2.076, CI 1.543–1.047) showed a significant association with ARC risk under the recessive model. The association remained significant after correcting for multiple testing and adjusting for age and gender. A stratification analysis was also performed to explore the relationship between these SNPs and the different subtypes of ARC. Only one tag SNPs (rs6657114, *p* = 2.94 × 10^−5^, OR 3.295, CI 1.883–5.765) were identified as a significant risk factor for cortical cataract under the recessive model.

**Table 3:** The relationship between *GJA8* tag SNPs and ARC risk

SNP	Genetic model	*χ* ^2^ test (*p*/*p*_a_)	Logistic regression	
			*p*/*p*_a_	OR (95% CI)
rs6657114	Dominant	0.067	–	–
	Recessive	0.002	–	–
	Additive	0.005	–	–
rs6688578	Dominant	0.062	–	–
	Recessive	0.978	–	–
	Additive	0.166	–	–
rs7544630	Dominant	0.428	–	–
	Recessive	0.522	–	–
	Additive	0.669	–	–
rs1532399	Dominant	0.053	–	–
	Recessive	0.003	–	–
	Additive	0.008	–	–
rs2132397	Dominant	4.50 × 10^−5^/7.70 × 10^−3^	1.68 × 10^−4^/0.029	1.638 (1.267–2.118)
	Recessive	0.156	–	–
	Additive	2.25 × 10^−4^/0.038	5.41 × 10^−5/^/9.25 × 10^−3^	1.413 (1.195–1.671)
rs7541950	Dominant	0.093	–	–
	Recessive	1.20 × 10^−5^/2.05 × 10^−3^	1.36 × 10^−6^/2.33 × 10^−4^	2.076 (1.543–1.047)
	Additive	7.00 × 10^−5^/0.012	1.78 × 10^−4^/0.030	1.543 (1.230–1.935)
rs9437981	Dominant	0.074	–	–
	Recessive	0.004	–	–
	Additive	0.011	–	–
rs6674829	Dominant	0.216	–	–
	Recessive	0.003	–	–
	Additive	0.011	–	–

### The alteration of autophagy in HLE cells caused by GJA8

**Fig. 1:**
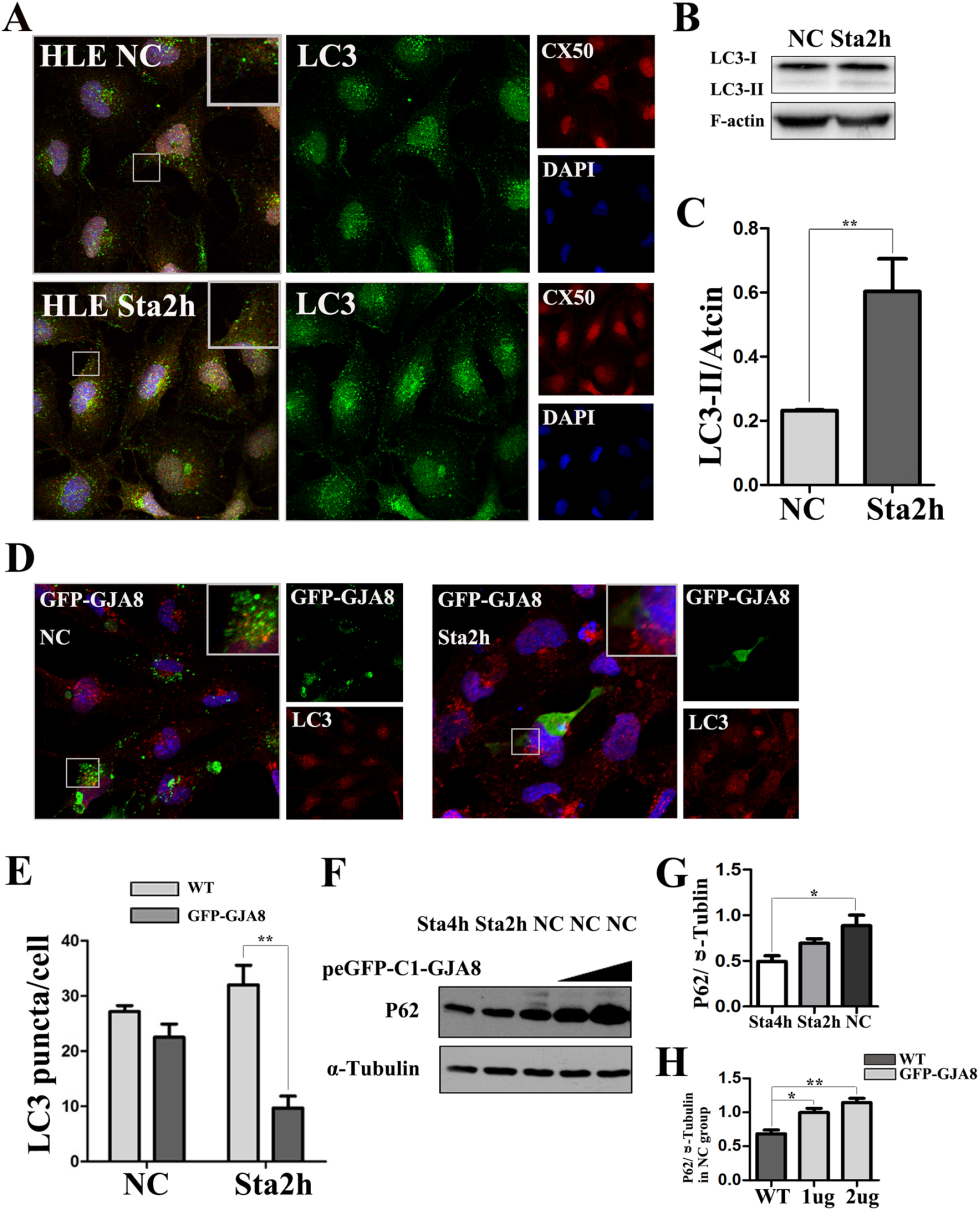
Overexpressed GJA8 protein modulated the level of LC3 and P62 in HLE cells. **a** HLE cells stained for GJA8 (red) and LC3 (green) in the NC and Sta2h groups. **b** Western blot with LC3-I/II in NC and Sta2h groups in HLE cells. **c** The average level of LC3-II/Actin of three independent experiments. **d** Transfected with peGFP-C1-GJA8 in the NC and Sta2h groups, HLE cells stained for LC3 (red). **e** Mean number of LC3 puncta for each treatment (*n* = 3 wells, 3 independent experiments, > 50 cells per experiment). **f** HLE cells for corresponding treatment and transfected with peGFP-C1-GJA8 for 0ug or 1ug or 2ug, testing the expression of P62 and α-Tubulin. **g** The average level of P62/α-Tubulin of three independent experiments in Sta4h/Sta2h/NC group. **h** The average level of P62/α-Tubulin of three independent experiments transfected with peGFP-C1-GJA8 for 0ug or 1ug or 2ug in NC group. All values are represented as the mean + SEM; *p < 0.05, **p < 0.01 indicate significant differences with corresponding groups. Nuclei are stained with DAPI

To prove whether starvation can induce autophagy to detectable levels in HLE cells, the alteration of the autophagic protein LC3, which is necessary in the elongation and formation process of autophagosome, therefore used to represent the level of autophagy (Jiang and Mizushima 2015), was tested under both normal and starved conditions via immunofluorescence and western blot (Fig. 1a, b), and the results revealed that the LC3-II were significantly increased under starved conditions (Fig. 1c). LC3 puncta were detected after transfecting peGFP-C1-GJA8 plasmid into HLE cells via immunofluorescence. And it was found that the LC3 puncta decreased in GJA8-overexpressed HLE cells, even under starved conditions (Fig. 1d, e). To further confirm these results, this study also detected the expression of P62, which recruited by LC3 and involved in the elongation process, and negatively related with the level of autophagy (Jiang and Mizushima 2015; Pankiv et al. 2007), via western blot (Fig. 1f). In NC group, a significantly higher expression of P62 was detected after transfected with 1.0 and 2.0 ug peGFP-C1-GJA8 plasmid (Fig. 1h).

## Discussion

The GJA8 protein is the major component of gap junction channels and hemichannels in the lens (Beyer et al. 2013; Goodenough 1992). To date, no study has thoroughly explored the relationship between the *GJA8*-tagged SNPs and ARC. Thus, this study intends to uncover the association between *GJA8*-tagged SNPs and ARC in the Chinese population. In the present study, a potential relationship between three *GJA8*-tagged SNPs (rs2132397, rs7541950 and rs6657114) and ARC is identified. Moreover, using immunofluorescence and western blot, it is found that GJA8 is involved in autophagy in HLE cells, which indicates another mechanism by which genetic variations in *GJA8* could lead to cataract formation.

**Fig. 2:**
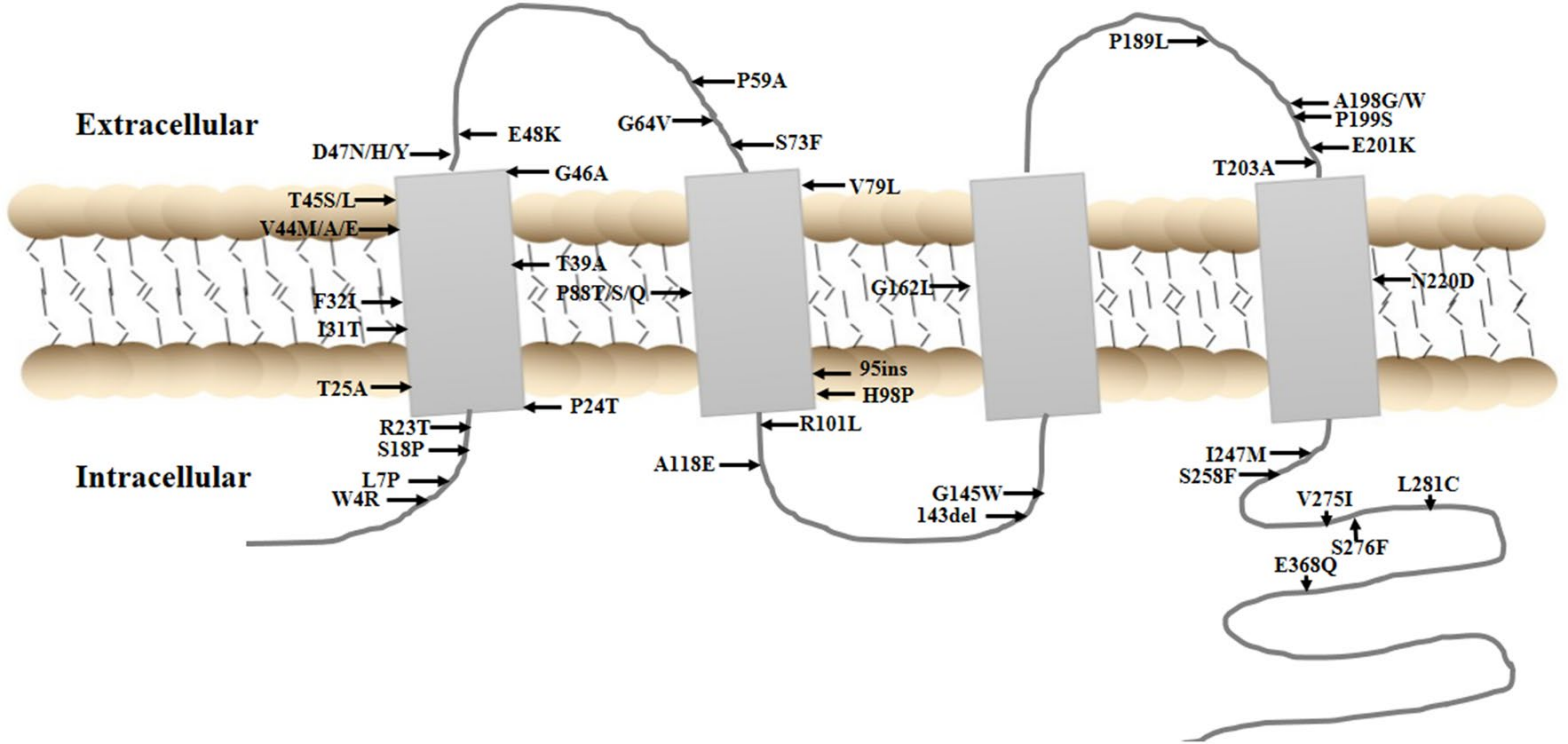
Schematic diagram of GJA8 reported variants in Pubmed database

As mentioned above, the *GJA8* gene has always been considered a cataract-related gene. More than 20 *GJA8* mutations have been detected in congenital cataract pedigrees of different populations thus far (Beyer et al. 2013; Dang et al. 2016; Goodenough 1992; Graw et al. 2009; Jiang 2010; Li et al. 2013; Liu et al. 2011; Patel et al. 2017; Ren et al. 2017; Shiels and Hejtmancik 2013; Zhao et al. 2017; Zhou et al. 2011; Zhu et al. 2014). However, few studies have reported the relationship of *GJA8* mutations with ARC. In general, variants in lens proteins, which would cause protein to aggregate rapidly and directly, tend to result in congenital cataracts. In contrast, variants that merely increase susceptibility to environmental risk factors usually contribute to age-related cataracts (Shiels 2017). It is worth noting that GJA8 variants are risk factors for both types of cataracts. Moreover, GJA8 variants related to congenital cataracts could occur in any domain of the GJA8 protein (Fig. 2, Supplementary Table S1).

In the present study, for total ARC, rs2132397 and rs7541950 are found to confer 1.41- and 1.54-fold increased risks under the additive model, respectively. Also, rs2132397 is found to increase ARC risk under the dominant model, and rs7541950 is found to confer a 2.08-fold increased ARC risk under the recessive model. In addition, for ARC subtypes, only rs6657114 is found to be a potential risk factor for cortical cataract under the recessive model. Yet, for congenital individuals with *GJA8* variants, nuclear cataract is the most common subtype. The results of this study indicate that *GJA8* variants may induce diverse cataract subtypes. Additionally, the rs2132397 is located in the 3′-terminal untranslated region (3′-UTR) region of *GJA8*, which indicates its potential role in the interference of translation initiation. The functional consequences of the other two SNPs (rs754195 and rs6657114) are still unknown, as these two polymorphisms are located in a non-coding intron. This study speculates that they may be in linkage disequilibrium with the actual disease-causing variants in *GJA8*, or even in a nearby causative gene. The data expand the spectrum of *GJA8* variants and cataract phenotypes; however, the genotype–phenotype correlation remains unclear.

For the avascular lens, lens cell survival is dependent on intercellular communication through an extensive network of gap junctions (Ren et al. 2017). Generally, gap junction channels in HLE cells consist of two connexons; one connexon comprises one hexamer of connexins, also called a hemichannel, which is mainly formed by three different connexin forms (GJA3, GJA8, and GJA1) (Beyer et al. 2013; Goodenough 1992; Zhu et al. 2014). The team for this study has reported impaired gap junctions in HLE cells with *GJA8* mutations (p.V44A and p.D47H, identified from two congenital cataract families) (Li et al. 2013; Zhu et al. 2014), which is in accordance with other studies and is considered to be the main pathogenesis of congenital cataract patients with *GJA8* mutations (Shiels and Hejtmancik 2013). However, several studies found that, in some congenital families with *GJA8* mutations, the gap junction channel and hemichannel functions were only mildly impaired, which was insufficient to cause congenital cataracts (Graw et al. 2009). In addition, mutations in *GJA8* could result in different lens morphologies and diverse patterns of lens opacities under different genetic models (Dang et al. 2016; Li et al. 2013; Patel et al. 2017; Ren et al. 2017; Zhao et al. 2017; Zhou et al. 2011). Therefore, other underlying molecular mechanisms may exist.

Autophagy is an evolutionarily conserved process that is essential for cell survival and development (Sagona et al. 2014). In lens development, autophagy participates in the degradation of cellular organelles and proteins and is crucial for transparent lens formation (Morishita and Mizushima 2016). It is worth noting that more than 80% of autophagosomes contain connexin proteins (Bejarano et al. 2014). Bejarano et al. found that connexins can form a Cx-Atg complex (an important complex involved in the initial step of intracellular autophagy) to modulate the formation of the autophagosomes through disturbing the formation of the complex ATG5/ATG16L/ATG12 (Bejarano et al. 2014). Lichtenstein et al. reported that as the GJA8 mutation was not enclosed by specific structures containing the autophagy-related protein LC3, resulting in accumulation in the cytoplasm (Lichtenstein et al. 2011). Thus, this study speculates that autophagy is important for the normal turnover of GJA8 protein, which may reveal another novel mechanism for cataract formation caused by GJA8 mutation accumulation. To prove this, immunofluorescence and western blot are used to detect the alteration of the autophagic proteins LC3 and P62 in different situations as well as in normal HLE cells or overexpressed GJA8 HLE cells. LC3 is reported to be necessary in the elongation and formation process of autophagosome, which is therefore usually used to represent the level of autophagy (Jiang and Mizushima 2015). P62, recruited by LC3 and also involved in the elongation process, could be degraded by autophagosome (Pankiv et al. 2007). Thus, the level of P62 is negatively related with the level of autophagy (Jiang and Mizushima 2015; Pankiv et al. 2007). The present study finds that, in GJA8-overexpressed HLE cells, LC3 puncta decreased even under starvation conditions. which revealed overexpressed GJA8 negatively affect the process of autophagy. Consistent with this, the expression of P62 increased in GJA8-overexpressed HLE cells in NC group, compared with no transfection NC group, and this tendency was increased as the transfected with peGFP-C1-GJA8 plasmid increased. These results indicate that the overexpressed GJA8 protein alters the level of LC3 and P62, which suggests that GJA8 may be involved in the process of autophagy.

The current study provides new evidence to support the *GJA8* gene as a susceptibility gene for ARC in the Chinese population. In addition to interfering with the functions of gap junctions and hemichannels, *GJA8* variants may also induce lens opacification by disturbing the autophagic process. Given the limited sample size and single population, further studies in other, larger populations are warranted. The relationship between GJA8 and autophagy also needs to be addressed by elucidating the interactive molecules in detail in future research.

## Electronic supplementary material

Below is the link to the electronic supplementary material.

Supplementary material 1 (DOCX 24 KB)

Supplementary material 2 (DOCX 1339 KB)

## References

[CR1] Bejarano E, Yuste A, Patel B, Stout RF, Spray DC, Cuervo AM (2014). Connexins modulate autophagosome biogenesis. Nat Cell Biol.

[CR2] Beyer EC, Ebihara L, Berthoud VM (2013). Connexin mutants and cataracts. Front Pharmacol.

[CR3] Bourne RR, Stevens GA, White RA, Smith JL, Flaxman SR, Price H, Jonas JB, Keefe J, Leasher J, Naidoo K, Pesudovs K, Resnikoff S, Taylor HR, Vision Loss Expert (2013). Causes of vision loss worldwide, 1990–2010: a systematic analysis. Lancet Glob Health.

[CR4] Chylack LT, Leske MC, McCarthy D, Khu P, Kashiwagi T, Sperduto R (1989). Lens opacities classification system II (LOCS II). Arch Ophthalmol.

[CR5] Congdon NG, Friedman DS, Lietman T (2003). Important causes of visual impairment in the world today. JAMA.

[CR6] Dang FT, Yang FY, Yang YQ, Ge XL, Chen D, Zhang L, Yu XP, Gu F, Zhu YH (2016). A novel mutation of p.F32I in GJA8 in human dominant congenital cataracts. Int J Ophthalmol.

[CR7] Goodenough DA (1992). The crystalline lens. A system networked by gap junctional intercellular communication. Semin Cell Biol.

[CR8] Graw J, Schmidt W, Minogue PJ, Rodriguez J, Tong JJ, Klopp N, Illig T, Ebihara L, Berthoud VM, Beyer EC (2009). The GJA8 allele encoding CX50I247M is a rare polymorphism, not a cataract-causing mutation. Mol Vis.

[CR9] Hammond CJ, Snieder H, Spector TD, Gilbert CE (2000). Genetic and environmental factors in age-related nuclear cataracts in monozygotic and dizygotic twins. N Engl J Med.

[CR10] Hammond CJ, Duncan DD, Snieder H, de Lange M, West SK, Spector TD, Gilbert CE (2001). The heritability of age-related cortical cataract: the twin eye study. Investig Ophthalmol Vis Sci.

[CR29] Ibaraki N, Chen SC, Lin LR, Okamoto H, Pipas JM, Reddy VN (1998). Human lens epithelial cell line. Exp Eye Res.

[CR11] Jiang JX (2010). Gap junctions or hemichannel-dependent and independent roles of connexins in cataractogenesis and lens development. Curr Mol Med.

[CR12] Jiang P, Mizushima N (2015). LC3- and p62-based biochemical methods for the analysis of autophagy progression in. Mamm Cells Methods.

[CR13] Klein BE, Klein R, Linton KL (1992). Prevalence of age-related lens opacities in a population: the Beaver Dam Eye Study. Ophthalmology.

[CR14] Li JY, Wang QW, Fu QY, Zhu YA, Zhai Y, Yu YH, Zhang K, Yao K (2013). A novel connexin 50 gene (gap junction protein, alpha 8) mutation associated with congenital nuclear and zonular pulverulent. Cataract Mol Vis.

[CR15] Lichtenstein A, Minogue PJ, Beyer EC, Berthoud VM (2011). Autophagy: a pathway that contributes to connexin degradation. J Cell Sci.

[CR16] Liu YY, Ke M, Yan M, Guo SR, Mothobi ME, Chen QA, Zheng F (2011). Association between gap junction protein-alpha 8 polymorphisms and age-related cataract. Mol Biol Rep.

[CR17] Mackay DS, Bennett TM, Culican SM, Shiels A (2014). Exome sequencing identifies novel and recurrent mutations in GJA8 and CRYGD associated with inherited cataract. Hum Genom.

[CR18] Morishita H, Mizushima N (2016). Autophagy in the lens. Exp Eye Res.

[CR19] Pankiv S, Clausen TH, Lamark T, Brech A, Bruum JA, Outzen H, Overvatn A, Bjorkoy G, Johansen T (2007). p62/SQSTM1 binds directly to Atg8/LC3 to facilitate degradation of ubiquitinated protein aggregates by autophagy. J Biol Chem.

[CR20] Patel R, Zenith RK, Chandra A, Ali A (2017). Novel mutations in the crystallin gene in age-related cataract patients from a north Indian population. Mol Syndromol.

[CR21] Ren M, Yang XG, Dang XJ, Xiao JA (2017). Exome sequencing identifies a novel mutation in GJA8 associated with inherited cataract in a Chinese family Graefes. Arch Clin Exp Ophthalmol.

[CR22] Sagona AP, Nezis IP, Stenmark H (2014). Association of CHMP4B and autophagy with micronuclei: implications for cataract formation. BioMed Res Int.

[CR23] Shiels HJF (2017). Mutations and mechanisms in congenital and age-related cataracts. Exp Eye Res.

[CR24] Shiels A, Hejtmancik JF (2013). Genetics of human cataract. Clin Genet.

[CR25] Su S, Yao Y, Zhu RR, Liang CK, Jiang SQ, Hu N, Zhou J, Yang M, Xing Q, Guan HJ (2013). The associations between single nucleotide polymorphisms of DNA repair genes, DNA damage, and age-related cataract: Jiangsu Eye Study. Investig Ophthalmol Vis Sci.

[CR26] Zhao Z, Fan Q, Zhou P, Ye H, Cai L, Lu Y (2017). Association of alpha A-crystallin polymorphisms with susceptibility to nuclear age-related cataract in a Han Chinese population. BMC Ophthalmol.

[CR27] Zhou Z, Wang B, Hu S, Zhang C, Ma X, Qi Y (2011). Genetic variations in GJA3, GJA8, LIM2, and age-related cataract in the Chinese population: a mutation screening study. Mol Vis.

[CR28] Zhu Y, Yu H, Wang W, Gong X, Yao K (2014). A novel GJA8 mutation (p.V44A) causing autosomal dominant congenital cataract. PLoS One.

