# Supplementary material for: The impact of GJA8 SNPs on susceptibility to age-related cataract
Source: Hum Genet. 2018 Oct 22;137(11):897–904. doi: 10.1007/s00439-018-1945-5 (PMC6267713; doi:10.1007/s00439-018-1945-5)
Supplement: Supplementary file 1 — Supplementary material 1 (DOCX 24 KB) [file 439_2018_1945_MOESM1_ESM.docx]

**Table S1****:**

| **Author** | **Publication Year** | **Country** | **GJA8 Variants** | **Cataract Type** |
| --- | --- | --- | --- | --- |
| Cui et al. | 2018 | China | p.H95_A96insYAVHY | Congenital  cataract |
| Zhang et al. | 2018 | China | c.130G>A p.V44M | Congenital  cataract |
| Zhang et al. | 2018 | China | c.10T>A p.W4R | Congenital  cataract |
| Micheal et al. | 2018 | Pakistan | c.53C>T p.S18P  c.175C>G p.P59A | Congenital  cataract |
| Qiao et al. | 2018 | China | c.426_440delGCTGGAGGGGACCCT p.143_147delLEGTL | Congenital  cataract |
| Patel et al. | 2017 | Indian | \| g.147380435C>A p.A118E \|  \| \| --- \| --- \| | Age-related cataract |
| Javadiyan et al. | 2017 | Australia | c.73T＞C p.T25A；  c.484G＞A p.G162L;  c.134G＞C p.T45S | Congenital  cataract |
| Mohebi et al. | 2017 | Iran | c.130G>A p.V44M  c.301G>T p.R101L  c.134G>T p.W45L | Congenital  cataract |
| Shen et al. | 2017 | China | c.139G>A p. D47N | Congenital  cataract |
| Ren et al. | 2017 | China | c.433G > T p.G145W | Congenital  cataract |
| Kuo et al. | 2017 | USA | c.658A>G p.N220D | Congenital  cataract |
| Dang et al. | 2016 | China | c.94T>C p.F32I | Congenital  cataract |
| Min et al. | 2016 | China | c.426_440delGCTGGAGGGGACCCT p.143_147delLEGTL | Congenital  cataract |
| Kumar et al. | 2016 | USA | c.1102G>C p.E368Q | Congenital  cataract |
| Yang et al. | 2015 | China | c.218C>T p. S73F | Congenital  cataract |
| Liang et al. | 2015 | China | c.139G>A p.D47N | Congenital  cataract |
| Ge et al. | 2014 | China | c.264 C>A p.P88T | Congenital  cataract |
| Zhu et al. | 2014 | China | c.131T>C p.V44A | Congenital  cataract |
| Mackay et al. | 2014 | USA | c.20T > C p.L7P  c.293A>C p.H98P | Congenital  cataract |
| Prokudin et al. | 2014 | Australia | c.593G4A p.A198G | Congenital  cataract |
| Chen et al. | 2014 | China | c.773C4T p.S258F | Congenital  cataract |
| Su et al. | 2013 | China | c.601G>A p.E201K | Congenital  cataract |
| Li et al. | 2013 | China | c.139G>C p.D47H | Congenital  cataract |
| Ponnam et al. | 2013 | India | c.658C>T p.P199S | Congenital  cataract |
| Wang et al. | 2011 | China | c.139G>A p.D47N | Congenital  cataract |
| Liu et al. | 2011 | China | rs1495960;  rs9437983 | Age-related cataract |
| Yang et al. | 2011 | China | c.139G>C p.D47H | Congenital  cataract |
| Zhou et al. | 2011 | China | c.823G>A p.V275I | Age-related cataract |
| Kumar et al. | 2011 | India | c.T905C p.L281C | Congenital  cataract |
| Sun et al. | 2011 | China | c.136G>A p.G46A  c.116C>G p.T39A | Congenital  cataract |
| Roshan et al. | 2010 | Germany | c.70C>A p. P24T | Congenital  cataract |
| Hu et al. | 2010 | China | c.592C>T p.R198W | Congenital  cataract |
| Gao et al. | 2010 | China | c.773C>T S258F | Congenital  cataract |
| Wang et al. | 2009 | China | c.92T>C p.I31T | Congenital  cataract |
| Yan et al. | 2009 | China | c.827 C>T p.S276F | Congenital  cataract |
| [Ponnam](https://www.ncbi.nlm.nih.gov/pubmed/?term=Ponnam%20SP%5BAuthor%5D&cauthor=true&cauthor_uid=21720542) et al. | 2009 | India | c.670insA; p.T203AsnfsX47 | Congenital  cataract |
| Arora et al. | 2008 | Britain | c. 139G>C p.D47H | Congenital  cataract |
| Vanita et al. | 2008 | Germany | c.262C>A p.S276F | Congenital  cataract |
| Lin et al. | 2008 | China | c.139G>T p.D47Y | Congenital  cataract |
| Vanita et al. | 2008 | Germany | c.134G>C p.W45S | Congenital  cataract |
| Yan et al. | 2008 | China | c.262C>T p.P88S | Congenital  cataract |
| Schmidt et al. | 2008 | Germany | c.776insG | Congenital  cataract |
| Ponnam et al. | 2007 | India | c.670insA; p.T203AsnfsX47 | Congenital  cataract |
| Hansen et al. | 2007 | USA | P189L;V44E; R198Q | Congenital  cataract |
| Arora et al. | 2006 | Britain | c.262C>A p.P88Q | Congenital  cataract |
| Vanita et al. | 2006 | Germany | c.235G>C p.V79L | Congenital  cataract |
| Devi et al. | 2006 | India | c.593G>A p.R198Q;  c.131T>A p.V44E | Congenital  cataract |
| Zheng et al. | 2005 | China | c.191G>T p.G64V | Congenital  cataract |
| Willoughby et al. | 2003 | Iran | c.68G>C p.R23T | Congenital  cataract |
| Polyako et al. | 2001 | Rusia | c.741G>T p.I247M | Congenital  cataract |
| Berry et al. | 1999 | USA | c.142A>G p.E48K | Congenital  cataract |
| Shiels et al. | 2007 | USA | c.262C>T p.P88S | Congenital  cataract |

**Figure Legend:**

Table S1:Summary of GJA8 variants reported in Pubmed up to August 27, 2018

**References:**

Arora A, Minogue PJ, Liu X, Addison PK, Russel-Eggitt I, Webster AR, Hunt DM, Ebihara L, Beyer EC, Berthoud VM, Moore AT (2008) A novel connexin50 mutation associated with congenital nuclear pulverulent cataracts. J Med Genet 45:155-60

Arora A, Minogue PJ, Liu X, Reddy MA, Ainsworth JR, Bhattacharya SS, Webster AR, Hunt DM, Ebihara L, Moore AT, Beyer EC, Berthoud VM (2006) A novel GJA8 mutation is associated with autosomal dominant lamellar pulverulent cataract: further evidence for gap junction dysfunction in human cataract. J Med Genet 43(1):e2

Berry V, Mackay D, Khaliq S, Francis PJ, Hameed A, Anwar K, Mehdi SQ, Newbold RJ, Ionides A, Shiels A, Moore T, Bhattacharya SS (1999) Connexin 50 mutation in a family with congenital "zonular nuclear" pulverulent cataract of Pakistani origin. Hum Genet 105:168-70

Chen JH, Qiu J, Chen H, Pang CP, Zhang M (2014) Rapid and cost-effective molecular diagnosis using exome sequencing of one proband with autosomal dominant congenital cataract. Eye (Lond) ;28:1511-6. doi: 10.1038/eye.2014.158

Cui X, Zhou Z, Zhu K, Feng R, Han J, Li M, Wang S, Li J, Zhang J, Jiang Q, Zhang W, Mu H, Liu Y, Hu Y (2018) DNA Cell Biol 37:449-456. doi: 10.1089/dna.2017.4051

Dang FT, Yang FY, Yang YQ, Ge XL, Chen D, Zhang L, Yu XP, Gu F, Zhu YH A (2016) novel mutation of p.F32I in GJA8 in human dominant congenital cataracts. Int J Ophthalmol 9:1561-1567

Devi RR, Vijayalakshmi P (2006) Novel mutations in GJA8 associated with autosomal dominant congenital cataract and microcornea. Mol Vis ;12:190-5

Ge XL, Zhang Y, Wu Y, Lv J, Zhang W, Jin ZB, Qu J, Gu F. (2014) Identification of a novel GJA8 (Cx50) point mutation causes human dominant congenital cataracts. Sci Rep. 4:4121. doi: 10.1038/srep04121

Gao X, Cheng J, Lu C, Li X, Li F, Liu C, Zhang M, Zhu S, Ma X (2010) A novel mutation in the connexin 50 gene (GJA8) associated with autosomal dominant congenital nuclear cataract in a Chinese family. Curr Eye Res 35:597-604. doi: 10.3109/02713681003725831

Hansen L, Yao W, Eiberg H, Kjaer KW, Baggesen K, Hejtmancik JF, Rosenberg T (2007) Genetic heterogeneity in microcornea-cataract: five novel mutations in CRYAA, CRYGD, and GJA8. Invest Ophthalmol Vis Sci ;48:3937-44

Hu S, Wang B, Zhou Z, Zhou G, Wang J, Ma X, Qi Y (2010) A novel mutation in GJA8 causing congenital cataract-microcornea syndrome in a Chinese pedigree. Mol Vis 16:1585-92

Javadiyan S, Craig JE, Souzeau E, Sharma S, Lower KM, Mackey DA, Staffieri SE, Elder JE, Taranath D, Straga T, Black J, Pater J, Casey T, Hewitt AW, Burdon KP (2017) High-Throughput Genetic Screening of 51 Pediatric Cataract Genes Identifies Causative Mutations in Inherited Pediatric Cataract in South Eastern Australia. G3 (Bethesda);7:3257-3268. doi: 10.1534/g3.117.300109

Kumar M, Agarwal T, Khokhar S, Kumar M, Kaur P, Roy TS, Dada R (2011) Mutation screening and genotype phenotype correlation of α-crystallin, γ-crystallin and GJA8 gene in congenital cataract. Mol Vis ;17:693-707

Kuo DS, Sokol JT, Minogue PJ, Berthoud VM, Slavotinek AM, Beyer EC, Gould DB (2017) Characterization of a variant of gap junction protein α8 identified in a family with hereditary cataract. PLoS One;12:e0183438. doi: 10.1371/journal.pone.0183438

Li J, Wang Q, Fu Q, Zhu Y, Zhai Y, Yu Y, Zhang K, Yao K (2013) A novel connexin 50 gene (gap junction protein, alpha 8) mutation associated with congenital nuclear and zonular pulverulent cataract. Mol Vis 19:767-74

Lin Y, Liu NN, Lei CT, Fan YC, Liu XQ, Yang Y, Wang JF, Liu B, Yang ZL (2008) A novel GJA8 mutation in a Chinese family with autosomal dominant congenital cataract. Zhonghua Yi Xue Yi Chuan Xue Za Zhi ;25:59-62

Liang C, Liang H, Yang Y, Ping L, Jie Q (2015) Mutation analysis of two families with inherited congenital cataracts. Mol Med Rep ;12:3469-3475. doi: 10.3892/mmr

Liu Y, Ke M, Yan M, Guo S, Mothobi ME, Chen Q, Zheng F (2011) Association between gap junction protein-alpha 8 polymorphisms and age-related cataract. Mol Biol Rep 38:1301-7. doi: 10.1007/s11033-010-0230-z

Micheal S, Niewold ITG, Siddiqui SN, Zafar SN, Khan MI, Bergen AAB (2018) Delineation of Novel Autosomal Recessive Mutation in GJA3 and Autosomal Dominant Mutations in GJA8 in Pakistani Congenital Cataract Families. Genes (Basel);9. pii: E112. doi: 10.3390/genes9020112

Min HY, Qiao PP; Asan, Yan ZH, Jiang HF, Zhu YP, Du HQ, Li Q, Wang JW, Zhang J, Sun J, Yi X, Yang L (2016) Targeted Genes Sequencing Identified a Novel 15 bp Deletion on GJA8 in a Chinese Family with Autosomal Dominant Congenital Cataracts. Chin Med J (Engl) ;129:860-7. doi: 10.4103/0366-6999.178966

Mohebi M, Chenari S, Akbari A, Ghassemi F, Zarei-Ghanavati M, Fakhraie G, Babaie N, Heidari M (2017) Mutation analysis of connexin 50 gene among Iranian families with autosomal dominant cataracts. Iran J Basic Med Sci ;20:288-293. doi: 10.22038/IJBMS

Mackay DS, Bennett TM, Culican SM, Shiels A (2014) Exome sequencing identifies novel and recurrent mutations in GJA8 and CRYGD associated with inherited cataract. Hum Genomics ;8:19. doi: 10.1186/s40246-014-0019-6

[Patel R](https://www.ncbi.nlm.nih.gov/pubmed/?term=Patel%20R%5BAuthor%5D&cauthor=true&cauthor_uid=28690483), [Zenith RK](https://www.ncbi.nlm.nih.gov/pubmed/?term=Zenith%20RK%5BAuthor%5D&cauthor=true&cauthor_uid=28690483), [Chandra A](https://www.ncbi.nlm.nih.gov/pubmed/?term=Chandra%20A%5BAuthor%5D&cauthor=true&cauthor_uid=28690483), [Ali A](https://www.ncbi.nlm.nih.gov/pubmed/?term=Ali%20A%5BAuthor%5D&cauthor=true&cauthor_uid=28690483). Patel R, Zenith RK, Chandra A, Ali A (2017) Novel Mutations in the Crystallin Gene in Age-Related Cataract Patients from a North Indian Population. Mol Syndromol. 2017 8:179-186. doi: 10.1159/000471992

Ponnam SP, Ramesha K, Tejwani S, Ramamurthy B, Kannabiran C (2007) Mutation of the gap junction protein alpha 8 (GJA8) gene causes autosomal recessive cataract. J Med Genet ;44(7):e85

Ponnam SP, Ramesha K, Matalia J, Tejwani S, Ramamurthy B, Kannabiran C (2013) Mutational screening of Indian families with hereditary congenital cataract. Mol Vis ;19:1141-8

Polyakov AV, Shagina IA, Khlebnikova OV, Evgrafov OV (2001) Mutation in the connexin 50 gene (GJA8) in a Russian family with zonular pulverulent cataract. Clin Genet ;60:476-8

Ponnam SP, Ramesha K, Tejwani S, Ramamurthy B, Kannabiran C (2009) Mutation of the gap junction protein alpha 8 (GJA8) gene causes autosomal recessive cataract. BMJ Case Rep 2009. pii: bcr06.2009.1995. doi: 10.1136/bcr.06.2009.1995

Prokudin I, Simons C, Grigg JR, Storen R, Kumar V, Phua ZY, Smith J, Flaherty M, Davila S, Jamieson RV (2014) Exome sequencing in developmental eye disease leads to identification of causal variants in GJA8, CRYGC, PAX6 and CYP1B1. Eur J Hum Genet ;22:907-15. doi: 10.1038/ejhg.2013.268

Ren M, Yang XG, Dang XJ, Xiao JA (2017) Exome sequencing identifies a novel mutation in GJA8 associated with inherited cataract in a Chinese family. Graefes Arch Clin Exp Ophthalmol 255:141-151. doi: 10.1007/s00417-016-3513-9

Roshan M, Vijaya PH, Lavanya GR, Shama PK, Santhiya ST, Graw J, Gopinath PM, Satyamoorthy K (2010) A novel human CRYGD mutation in a juvenile autosomal dominant cataract. Mol Vis 16:887-96

Shen C, Wang J, Wu X, Wang F, Liu Y, Guo X, Zhang L, Cao Y, Cao X, Ma H (2017) Next-generation sequencing for D47N mutation in Cx50 analysis associated with autosomal dominant congenital cataract in a six-generation Chinese family. BMC Ophthalmol ;17:73. doi: 10.1186/s12886-017-0476-5

Senthil Kumar G, Dinesh Kumar K, Minogue PJ, Berthoud VM, Kannan R, Beyer EC, Santhiya ST (2016) The E368Q Mutant Allele of GJA8 is Associated with Congenital Cataracts with Intrafamilial Variation in a South Indian Family. Open Access J Ophthalmol ;1. pii: 106

Su D, Yang Z, Li Q, Guan L, Zhang H, E D, Zhang L, Zhu S, Ma X (2013) Identification and functional analysis of GJA8 mutation in a Chinese family with autosomal dominant perinuclear cataracts. PLoS One 8:e59926. doi: 10.1371/journal.pone.0059926

Sun W, Xiao X, Li S, Guo X, Zhang Q (2011) Mutational screening of six genes in Chinese patients with congenital cataract and microcornea. Mol Vis ;17:1508-13

Schmidt W, Klopp N, Illig T, Graw J (2008) A novel GJA8 mutation causing a recessive triangular cataract. Mol Vis 9;14:851-6.

Shiels A1, Mackay D, Ionides A, Berry V, Moore A, Bhattacharya S (1998) A missense mutation in the human connexin50 gene (GJA8) underlies autosomal dominant "zonular pulverulent" cataract, on chromosome 1q. Am J Hum Genet 62:526-32.

Vanita V, Singh JR, Singh D, Varon R, Sperling K (2008) A mutation in GJA8 (p.P88Q) is associated with "balloon-like" cataract with Y-sutural opacities in a family of Indian origin. Mol Vis 14:1171-5

Vanita V, Hennies HC, Singh D, Nürnberg P, Sperling K, Singh JR (2006) A novel mutation in GJA8 associated with autosomal dominant congenital cataract in a family of Indian origin. Mol Vis 12:1217-22

Vanita V1, Singh JR, Singh D, Varon R, Sperling K (2008) A novel mutation in GJA8 associated with jellyfish-like cataract in a family of Indian origin. Mol Vis 14:323-6

Wang K, Wang B, Wang J, Zhou S, Yun B, Suo P, Cheng J, Ma X, Zhu S (2009) A novel GJA8 mutation (p.I31T) causing autosomal dominant congenital cataract in a Chinese family. Mol Vis 15:2813-20

Wang L, Luo Y, Wen W, Zhang S, Lu Y (2011) Another evidence for a D47N mutation in GJA8 associated with autosomal dominant congenital cataract. Mol Vis 17:2380-5

Willoughby CE, Arab S, Gandhi R, Zeinali S, Arab S, Luk D, Billingsley G, Munier FL, Héon E (2003) A novel GJA8 mutation in an Iranian family with progressive autosomal dominant congenital nuclear cataract. J Med Genet 40:e124

Yan M, Zhou X, Chen YM, Ma JJ, Xiong CL, Cheng XH (2009) Analysis on gene mutations in a Chinese pedigree with autosomal dominant inheritance cataract. Zhonghua Yan Ke Za Zhi 45:693-8

Yang G, Xiong C, Li S, Wang Y, Zhao J (2011) A recurrent mutation in CRYGD is associated with autosomal dominant congenital coralliform cataract in two unrelated Chinese families. Mol Vis 17:1085-9

Yang Z, Li Q, Ma X, Zhu SQ (2015) Mutation analysis in Chinese families with autosomal dominant hereditary cataracts. Curr Eye Res ;40:1225-31. doi: 10.3109/02713683.2014.997885

Yan M, Xiong C, Ye SQ, Chen Y, Ke M, Zheng F, Zhou X (2008) A novel connexin 50 (GJA8) mutation in a Chinese family with a dominant congenital pulverulent nuclear cataract. Mol Vis 14:418-24

Zhang XH, Da Wang J, Jia HY, Zhang JS, Li Y1, Xiong Y, et al. (2018) Mutation profiles of congenital cataract genes in 21 northern Chinese families. [Mol Vis](https://www.ncbi.nlm.nih.gov/pmc/articles/PMC6054834/) 24:471-477

Zhang L, Liang Y, Zhou Y, Zeng H, Jia S, Shi J (2018) A Missense Mutation in GJA8 Encoding Connexin 50 in a Chinese Pedigree with Autosomal Dominant Congenital Cataract. Tohoku J Exp Med 244:105-111. doi: 10.1620/tjem.244.105

Zheng JQ, Ma ZW, Sun HM (2005) A heterozygous transversion of connexin 50 in a family with congenital nuclear cataract in the northeast of China. Zhonghua Yi Xue Yi Chuan Xue Za Zhi 22:76-8

Zhu Y, Yu H, Wang W, Gong X, Yao K (2014) A novel GJA8 mutation (p.V44A) causing autosomal dominant congenital cataract. PLoS One 9:e115406. doi: 10.1371/journal.pone.0115406

Zhou Z, Wang B, Hu S, Zhang C, Ma X, Qi Y (2011) Genetic variations in GJA3, GJA8, LIM2, and age-related cataract in the Chinese population: a mutation screening study. Mol Vis ;17:621-6
