# Supplementary material for: The impact of GJA8 SNPs on susceptibility to age-related cataract
Source: Hum Genet. 2018 Oct 22;137(11):897–904. doi: 10.1007/s00439-018-1945-5 (PMC6267713; doi:10.1007/s00439-018-1945-5)
Supplement: Supplementary file 2 — Supplementary material 2 (DOCX 1339 KB) [file 439_2018_1945_MOESM2_ESM.docx]

Supplementary Figure S1:


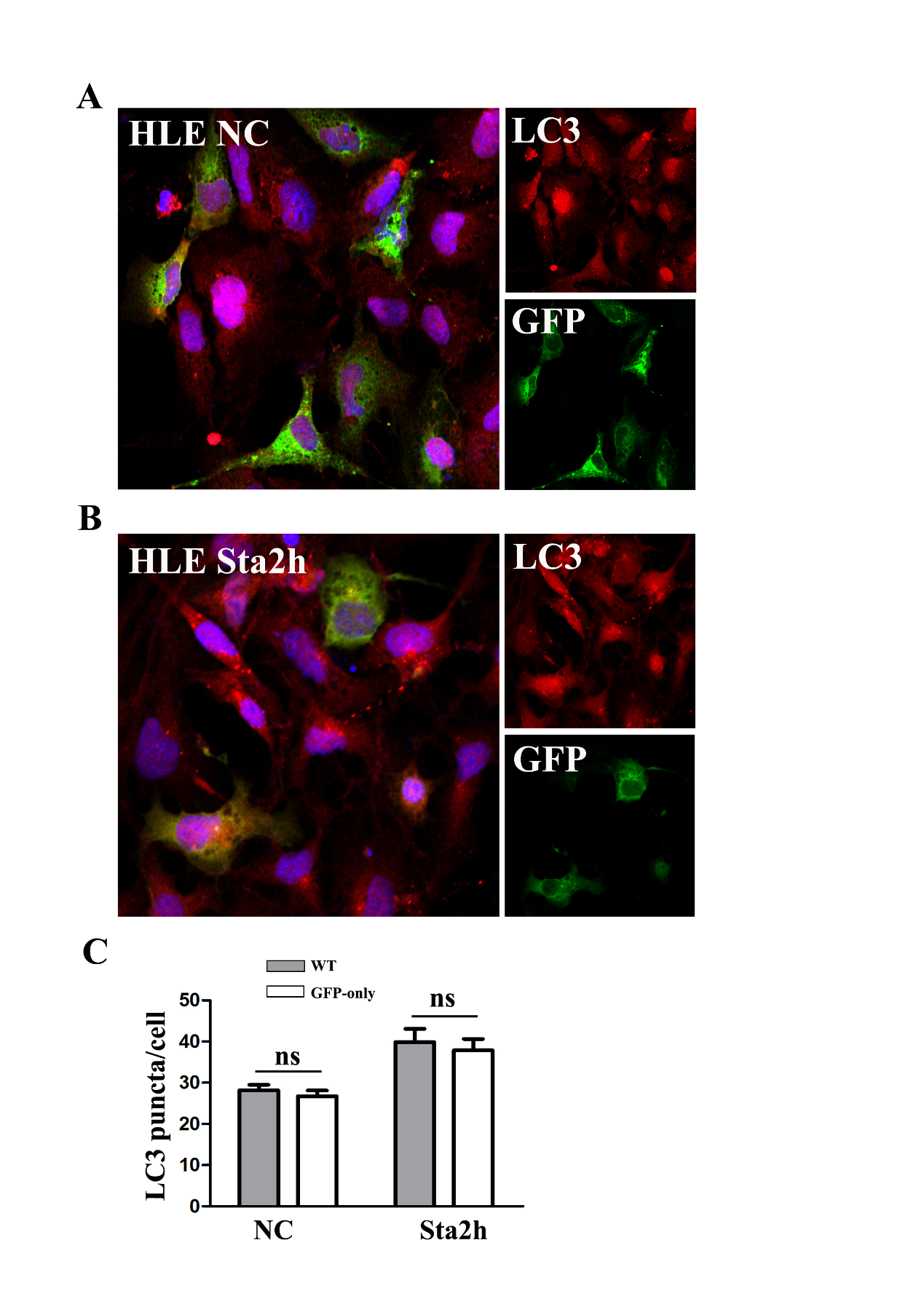


Figure legends:

Figure 1: (A) HLE cells transfected with GFP-only vector stained for LC3 (red) in the NC groups. (B) HLE cells transfected with GFP-only vector stained for LC3 (red) in the Sta2h groups. (C) Mean number of LC3 puncta of per cell for each treatment in HLE cells (n=3 wells, 3 independent experiments, >50 cells per experiment). All values are represented as the mean+SEM; NS: indicate no significant differences with corresponding groups. Nuclei are stained with DAPI.
